# The impact of activating an empathic focus during COVID19 on healthcare workers motivation for hand hygiene compliance in moments serving the protection of others: a randomized controlled trial study

**DOI:** 10.1007/s10389-022-01725-z

**Published:** 2022-06-20

**Authors:** Claudia Sassenrath, Svenne Diefenbacher, Viktoria Kolbe, Heide Niesalla, Johannes Keller

**Affiliations:** 1 Department of Social Psychology, Ulm University, Albert-Einstein Allee 47, 89069 Ulm, Germany; 2 Hartmann Science Center, Bode Chemie GmbH, Melanchthonstraße 27, 22525 Hamburg, Germany

**Keywords:** Hand hygiene compliance, Empathic focus, COVID-19 pandemic

## Abstract

**Aim:**

The “Five moments of hand hygiene” (World Health Organization 2009) can be classified into moments of hand hygiene before and after patient care. Based on research indicating that hand hygiene compliance differs with regard to moments before and after patient care, this research evaluates the effectiveness of an empathy-based intervention in motivating hand hygiene compliance with regard to moments before patient care which protect vulnerable individuals from contamination and infection.

**Subjects and method:**

An online experiment involving 68 healthcare professionals working at a German hospital during the first wave of the COVID-19 pandemic investigates whether instructing healthcare professionals to consider consequences for others (vs for themselves) if they contracted SARS-CoV-2 promotes hand hygiene compliance referring to moments before (vs after) patient care.

**Results:**

In the condition in which healthcare professionals considered consequences for others if they contracted SARS-CoV-2 (other-focus condition), ratings of importance increased (*M* = 3.49, *SD* = 1.30) compared to the condition in which healthcare professionals considered consequences for themselves (*M* = 2.68, *SD* = 1.24), *F*(1,66) = 6.87, *p* = .011, _part_η^2^ = .09. Participants in the *other-focus* condition reported more intentions to comply with “before moments” in the future (*M* = 3.34, *SD* = 1.14) compared to participants in the *self-focus* condition (*M* = 2.77, *SD* = 0.80), *F*(1,66) = 6.15, *p* = .016, _part_η^2^ = .09.

**Conclusion:**

Results indicate that activating an empathic focus in the context of the current pandemic promotes perceived importance and motivation of healthcare professionals to comply with moments aiming at protecting vulnerable others.

**Supplementary Information:**

The online version contains supplementary material available at 10.1007/s10389-022-01725-z.

## Introduction

Hand hygiene compliance by healthcare workers is vital for preventing healthcare-associated infections (HAIs) and also plays a significant role in preventing pathogen transmission such as COVID19 in hospitals (Gundersborg Sandbøl et al. 2022; World Health Organization (WHO) 2020). Nevertheless, compliance with hand hygiene guidelines is still alarmingly low in many healthcare institutions (Clancy et al. 2021). Therefore, the present study examined whether activating an empathic focus within this pandemic — by activating thoughts about the implications for *others* (vs for *themselves*) if participants contracted SARS-CoV-2 — fosters healthcare professionals’ hand hygiene compliance (HHC), particularly in contexts in which vulnerable others are protected by correctly performed hand hygiene behavior. This notion is based on the argument that empathy as an interpersonal orientation involves both an affective connection as well as adopting the perspective of other individuals (Batson 2009). This in turn leads to increased valuing of other individuals’ welfare and finally results in providing help and assistance (Batson 2009). Activating an empathic focus should therefore ultimately promote behavior that supports other individuals’ well-being. Hand hygiene behavior is a likely candidate in this context, because it represents a behavior clearly affecting others’ well-being, namely their state of health, to a considerable extent (Allegranzi and Pittet 2009). A recent meta-analysis indicates, for instance, that a single hand hygiene action is associated with a decrease in the daily likelihood of an acute respiratory infection (Mo et al. 2022).

Appropriate hand hygiene behavior in healthcare is exactly defined by evidence-based guidelines, precisely by the WHO (2009), which include the “Five moments of hand hygiene”. These guidelines describe in which types of situations and by what means hand hygiene behavior should be performed to interrupt pathogen transmission. The WHO’s five moments (WHO 2009) comprise “before patient contact” (Moment 1), “before aseptic procedure” (Moment 2), “after body fluid exposure risk” (Moment 3), “after patient contact” (Moment 4), and “after contact with patient surroundings” (Moment 5). Moments 1 and 2 represent the two “before moments”, which mainly serve the goal to prevent patients from colonization and infection. Moments 3, 4, and 5 represent the three “after moments” and are mainly concerned with preventing healthcare workers’ colonization and contamination (cf. Kingston et al. 2016). Consequently, hand hygiene behavior can be motivated by both the goal of protecting oneself and the goal of protecting others. Furthermore, recent research indicates that HHC differs with regard to the two types of moments, with lower compliance before patient contact than after. Specifically, Wetzker et al. (2016) provided data from German hospitals indicating that HHC before patient contact is lower (70%) compared to HHC after patient contact (82,5%). These findings are mirrored in a systemic review of published studies by Kingston et al. (2016), which also indicates that HHC is lower before patient contact (21%) compared to HHC after patient contact (47%). These findings are noteworthy given that patients are more vulnerable with regard to infections, as they can be weakened by their medical condition (immune status, wounds) and/or the treatment they receive. Moreover, it has been shown that invasive procedures (e.g., catheterization) are associated with an increased risk of HAIs (Schreiber et al. 2018). Consequently, patients probably benefit more (in absolute terms) from correctly performed disinfection measures than healthcare professionals, especially with regard to the “before moments”.

The development and testing of interventions aimed at increasing HHC involving “before moments” is, therefore, urgently needed. A promising approach in this context is the activation of an empathic focus in healthcare professionals. This assumption can be further substantiated when conceptualizing empathy as a basic moral emotional process fostering morality-motivated actions (Tangney et al. 2007). In the context of healthcare, morally appropriate actions mean that patients are saved from (further) suffering (e.g., by transmission of pathogens). Accordingly, one means of realizing this morally motivated inclination is the diligent observance of hand hygiene standards, especially with regard to the "before moments", which specifically serve to protect patients from the transmission of pathogens (Sax et al. 2007). Notably, previous research already demonstrated the positive impact of an empathy induction on healthcare workers’ *frequency* of hand hygiene behavior (Sassenrath et al. 2016). However, given that an increase in mere frequency might also include unnecessary hand disinfections, which may be detrimental because they unnecessarily consume valuable time (see Stahmeyer et al. 2017), the present study goes one step beyond. Here, we test the impact of activating an empathic focus within the present pandemic on healthcare workers hand hygiene *compliance*; that is, whether they are willing to more diligently adhere to guidelines defining when to perform a hand hygiene activity — particularly those guidelines aiming at protecting of vulnerable others.

Therefore, this study investigated whether instructing healthcare professionals to consider the consequences for *others* (compared to the consequences for *themselves*) if they became infected with SARS-CoV-2 leads to higher ratings of the importance of HHC in relation to 'before moments'. It was also tested whether this manipulation leads to increased self-reported behavioral intentions to perform appropriate hand hygiene in situations involving “before moments”.

We conducted an online rather than an on-site study, because the nationwide lockdown impeded entering hospitals or healthcare facilities during the study period (June–July 2020).

## Method

### Participants and design

Sixty-eight individuals who worked at a German municipal hospital of basic and regular care with more than 250 beds of nine medical, surgical, and interdisciplinary specialties, treating about 12.000 inpatients and 20.000 outpatients per year, participated in the study. Nurses were 78% of participants, 22% were physicians; 72 % of them had worked in their profession for 10 years or more; 19% took care of COVID-19 patients during the study period. All participants gave their consent to participate in a 10-minute online experiment with two experimental conditions. As compensation they received a voucher for 7.50 €, which could be redeemed in local stores and businesses. The study was approved by hospital management and employee representation. Participants were recruited by email via the hospital staff mailing list.

### Materials and procedure

Participants were recruited for a study addressing potential barriers to hand hygiene compliance in patient care during the COVID-19 pandemic. All instructions, tasks, and measures were presented using the software Unipark (https://www.unipark.com). By implementing a trigger, the experimental software ensured that all participants were randomly assigned to one of the two experimental conditions. Notably, a true randomization as implemented by the experimental software implies some variation and, thus, is associated with a not perfectly equal distribution of participants to the conditions (*self-focus* condition: *n* = 31; *other-focus* condition: *n* = 37). Table 1 depicts the distribution of participants’ profession (nurse, physician, or other), participants’ gender, and whether they had taken care of CVOID19 patients during the study period to the two conditions.

**Table 1:** Distribution of number of participants, participants’ profession, participants’ gender, and whether they took care of COVID19 patients during the study period between the two experimental conditions (*self-focus* condition vs *other-focus* condition)

	Self-focus condition	Other-focus condition
Number of participants	31	37
Profession		
Nurses Physicians Other (e.g., physiotherapist)	16 8 7	25 7 5
Participant gender		
Male female	12 19	11 26
Number of participants having taken care of COVID19 patients during study period	4	9

Hence, in the *self-focus* condition, the study started with asking participants to think about the consequences of contracting SARS-CoV-2 for *themselves*, both in terms of their personal health and their social and professional situation. In the *other-focus* condition, participants were asked to think about the consequences of contracting SARS-CoV-2 for *other individuals* with whom they were in contact. Participants should consider consequences for these individuals’ social and professional situation. In both conditions, participants were instructed to take notes.

Afterwards, participants in both experimental conditions indicated for each of the “Five moments of hand hygiene” (WHO 2009) how important they evaluate compliance with each moment, applying a Likert scale ranging from “1 = *not at all important*” to “7 = *very important*”. Subsequently, they indicated how difficult they viewed compliance with each of the five moments under current pandemic working conditions (Likert scale: “1 = *not at all difficult*” to “7 = *very difficult*”). Then, participants in both conditions made comparative judgments by indicating for which of the five moments they viewed compliance as most important. Next, participants answered the following item assessing behavioral intention in a comparative manner: “When thinking about the consequences of a SARS-CoV-2-infection for *others* (vs for *yourself*), which of the Five moments of hand hygiene do you intend to comply with most diligently during your work in the future?” by clicking on one of all five moments which were presented simultaneously at the screen.

Finally, as control variables, we assessed participants' perceived comprehensibility of the five moments (using a Likert scale from "1 = *not at all comprehensible*" to "7 = *completely comprehensible*"), and whether reducing the recommended exposure time from 30 to 15 seconds when using alcohol-based hand rubs would facilitate compliance with the five moments (using a Likert scale from "1 = *would not facilitate at all*" to "7 = *would facilitate significantly*"). Participants also rated their current working conditions according to how *pleasant*, *harmonious*, *uncomplicated*, *inspiring*, and *challenging* they perceived it. Additionally, participants answered the Empathic Concern subscale of the Interpersonal Reactivity Index (IRI, Davis 1983) and completed the Perceived Stress Scale (Cohen et al. 1983).

## Results

This study tested whether instructing healthcare professionals to consider the consequences for *others* (*other-focus* condition) if they contracted SARS-CoV-2 leads them to assign a higher importance to HHC indications involving “before moments” compared to participants imagining consequences for *themselves* (*self-focus* condition). Similarly, it was examined whether self-reported behavioral intention to perform appropriate hand hygiene in situations involving “before moments” is higher in the *other-focus* compared to the *self-focus* condition. Therefore, a multivariate ANOVA with the between-subjects factor (focus: self vs other) as independent variable and the reported absolute importance regarding each of the five moments as multivariate dependent variable revealed a significant main effect for the experimental factor, *F*(5,62) = 2.47, *p* = .042, _part_η^2^ = .17 (see Table 2). Likewise, when analyzing comparative judgments in which participants compared the importance of compliance between the five moments, participants indicated higher importance of compliance with “before moments” in the *other-focus* condition (*M* = 3.49, *SD* = 1.30) compared to the *self-focus* condition (*M* = 2.68, *SD* = 1.24), *F*(1,66) = 6.87, *p* = .011, _part_η^2^ = .09. Even more importantly, participants in the *other-focus* condition reported more intentions to comply with “before moments” in the future (*M* = 3.34, *SD* = 1.14) compared to participants in the *self-focus* condition (*M* = 2.77, *SD* = 0.80), *F*(1,66) = 6.15, *p* = .016, _part_η^2^ = .09 (see Table 2 for mean absolute importance ratings as well as for comparative importance and behavioral intention ratings).

**Table 2:** Mean values of importance and behavioral intention ratings regarding each of the “Five moments of hand hygiene” (WHO 2009) of *n* = 68 health care workers working at a German hospital during the first wave of the COVID-19 pandemic

	Self-focus condition	Other-focus condition
Mean importance rating Moment 1 (“before patient contact”)	6.4 (1.3)	7.0 (0.3)
Mean importance rating Moment 2 (“before aseptic procedure”)	6.6 (1.2)	6.7 (0.8)
Mean importance rating Moment 3 (“after body fluid exposure risk”)	6.9 (0.3)	6.9 (0.4)
Mean importance rating Moment 4 (“after patient contact”)	6.8 (0.5)	6.8 (0.9)
Mean importance rating Moment 5 (“after contact with patient surroundings”)	6.6 (0.7)	6.5 (1.0)
Mean comparative judgment regarding importance of “before moments”	2.7 (1.2)	3.5 (1.3)
Mean comparative judgment regarding behavioral intention to comply with “before moments”	2.8 (0.8)	3.3 (1.1)

Standard deviations are depicted in parenthesis

The experimentally induced focus (self vs other) did not affect any of the control variables (all *F*-values < 1). Also, including mean empathic concern and mean perceived stress as covariate into the analyses does not change results regarding comparative importance ratings as well as self-reported intentions regarding the “Five moments of hand hygiene”. However, it does slightly change the main effect of condition on absolute importance rating of each of the five moments from *F*(5,62) = 2.47, *p* = .042, _part_η^2^ = .17 to *F*(5,60) = 2.33, *p* = .053, _part_η^2^ = .16.

Likewise, including participants’ profession (nurse, physician, or other), participants’ gender and whether they had taken care of CVOID19 patients during the study period as control variable into analyses does not change results regarding comparative importance ratings as well as self-reported intentions regarding the “Five moments of hand hygiene”. However, it does slightly change the main effect of condition on absolute importance rating of each of the five moments from *F*(5,62) = 2.47, *p* = .042, _part_η^2^ = .17 to *F*(5,59) = 2.04, *p* = .086, _part_η^2^ = .15.

## Discussion

In summary, these findings indicate that empathy can be employed to foster the motivation in healthcare professionals to comply with hand hygiene guidelines, especially those aimed at protecting patients (i.e., Moment 1 ‘before patient contact’ and Moment 2 ‘before aseptic task’). Thereby, these findings bear relevant policy implications, as they suggest that it is not enough to simply activate thoughts about infectious diseases (such as COVID-19), for instance, by providing comprehensive information or by instructing healthcare professionals adequately. Instead, the focus on others is needed to increase compliance with evidence-based guidelines for appropriate hand hygiene that are particularly aiming at shielding patients from potentially lethal infections. This is noteworthy, given that numerous intervention programs have been developed to improve HHC without differentiating between “before moments“ and “after moments” (e.g., Ellingson et al. 2014), although compliance differs between these types (Kingston et al. 2016, Wetzker et al. 2016).

Notably, the present findings result from an online experiment using self-reports. Moreover, the experimental intervention (inducing an empathic focus in the context of the present pandemic) was realized only for a short time. Accordingly, it is helpful to replicate these findings using different empathy-inductions and also involving healthcare professionals from various healthcare institutions to test generalizability. As an additional limitation of this study, it should be noted that there was no condition including a “no-focus instruction”, which would have represented a baseline condition for the present outcomes. Also, we only assessed psychological determinants of compliant behavior and not compliant behavior itself, which certainly represents a limitation given well-known gaps between knowledge, behavioral intentions, and actual behavior (Sheeran and Webb 2016). Put differently, measuring potential long-term consequences of (repeated) empathy inductions and assessing their effect on actual HHC calls for on-site intervention studies in the future. Nevertheless, it should also be acknowledged that during nationwide lockdowns, assessing healthcare workers’ thoughts and self-reported motivations and intentions online by self-report is as close as a researcher can get when aiming at investigating this specific and highly relevant population during a global pandemic.

Taken together, the current findings highlight the usefulness of empathy in developing tailored interventions, pointing a fruitful path for future tools to be implemented in healthcare settings to enhance patient safety in public healthcare.

## Supplementary information

ESM 1 (SAV 50 kb)

ESM 2 (DOCX 97 kb)

## Data Availability

Data will be submitted as supplemental material.
