# Supplementary material for: The impact of activating an empathic focus during COVID19 on healthcare workers motivation for hand hygiene compliance in moments serving the protection of others: a randomized controlled trial study
Source: Z Gesundh Wiss. 2022 Jun 20:1–5. Online ahead of print. doi: 10.1007/s10389-022-01725-z (PMC9207872; doi:10.1007/s10389-022-01725-z)
Supplement: Supplementary file 2 — (DOCX 97 kb) [file 10389_2022_1725_MOESM2_ESM.docx]

Dear Participants,

First of all, we wish to thank you for your participation in this study in spite of the difficult current working situation which you are certainly facing.

The goal of this study is to assess potential barriers in the compliance of hand hygiene guidelines in the current pandemic situation, and how you think about these.

All individuals working in direct patient care at the XXX [name of the hospital] are invited to participated in this study.

Your participation is completely voluntary. All data is assessed completely anonymously and does not allow for any conclusion regarding who gave which answers. All assessed data is saved the University of Ulm and only the study team will have access to it.

Participation in the study will take about 5-8 minutes and as compensation we offer you a voucher over 7,50 Euro which can be redeemed at local stores and business in XXX [name of city where hospital is located]. T receive this voucher, we will redirect you to another website where you can enter your email address. Thereby, we can assure that your data from the study will not be saved together with your email address.

If you have any questions, please contact the principal investigator PD Dr. Claudia Sassenrath ([claudia.sassenrath@uni-ulm.de](mailto:claudia.sassenrath@uni-ulm.de)).

***Other-focus*** condition:

At the beginning of this study, we ask you to think about how it would be, if you became infect with the Corona-Virus SARS CoV-2. What consequences would your infection have for all the individuals that you are in contact with, for example, with regard to their health state but also regarding their working situation or their private life.

Please write down 2-3 sentences on your thoughts.

__________________________________________________________________________________

__________________________________________________________________________________

__________________________________________________________________________________

***Self-focus*** condition:

At the beginning of this study, we ask you to think about how it would be, if you became infect with the Corona-Virus SARS CoV-2. What consequences would your infection have for you, for example, with regard to your health state but also regarding your working situation or your private life.

Please write down 2-3 sentences on your thoughts.

__________________________________________________________________________________

__________________________________________________________________________________

__________________________________________________________________________________

The “5 Moments of hand hygiene” exactly define, when and in which moment during patient care you should disinfect your hands in order to prevent pathogen transmission:

We have listed the 5 moments here:

Moment 1 - before touching a patient

Moment 2 - before clean/aseptic procedure

Moment 3 - after a procedure or body fluid exposure risk

Moment 4 - after touching a patient

Moment 5 - after touching a patient's surroundings

***Other-focus*** condition:

Please indicate now, how important you deem the compliance with these five moments of hand hygiene when thinking about the consequences for others if you became infected with SARS CoV-2.

When thinking about the consequences for others if I became infected, I deem compliance with Moment 1 (before touching a patient) as

1 2 3 4 5 6 7

Not at all important very important

When thinking about the consequences for others if I became infected, I deem compliance with Moment 2 (before clean/aseptic procedure) as

1 2 3 4 5 6 7

Not at all important very important

When thinking about the consequences for others if I became infected, I deem compliance with Moment 3 (after a procedure or body fluid exposure risk) as

1 2 3 4 5 6 7

Not at all important very important

When thinking about the consequences for others if I became infected, I deem compliance with Moment 4 (after touching a patient) as

1 2 3 4 5 6 7

Not at all important very important

When thinking about the consequences for others if I became infected, I deem compliance with Moment 5 (after touching a patient's surroundings) as

1 2 3 4 5 6 7

Not at all important very important

***Self-focus*** condition:

Please indicate now, how important you deem the compliance with these five moments of hand hygiene when thinking about the consequences for yourself if you became infected with SARS CoV-2.

When thinking about the consequences for myself if I became infected, I deem compliance with Moment 1 (before touching a patient) as

1 2 3 4 5 6 7

Not at all important very important

When thinking about the consequences for myself if I became infected, I deem compliance with Moment 2 (before clean/aseptic procedure) as

1 2 3 4 5 6 7

Not at all important very important

When thinking about the consequences for myself if I became infected, I deem compliance with Moment 3 (after a procedure or body fluid exposure risk) as

1 2 3 4 5 6 7

Not at all important very important

When thinking about the consequences for myself if I became infected, I deem compliance with Moment 4 (after touching a patient) as

1 2 3 4 5 6 7

Not at all important very important

When thinking about the consequences for myself if I became infected, I deem compliance with Moment 5 (after touching a patient's surroundings) as

1 2 3 4 5 6 7

Not at all important very important

***Both*** conditions:

Please indicate now, how difficult you deem the compliance with these five moments of hand hygiene in your current working situation.

I deem compliance with Moment 1 (before touching a patient) in my current working situation as

1 2 3 4 5 6 7

Not at all difficult very difficult

I deem compliance with Moment 2 (before clean/aseptic procedure) as

1 2 3 4 5 6 7

Not at all difficult very difficult

I deem compliance with Moment 3 (after a procedure or body fluid exposure risk) as

1 2 3 4 5 6 7

Not at all difficult very difficult

I deem compliance with Moment 4 (after touching a patient) as

1 2 3 4 5 6 7

Not at all difficult very difficult

***Other-focus*** condition

When thinking about the consequences for others if you became infected, which of the “5 moments of hand hygiene” do you deem as most important when comparing them with each other?

Moment 1 - before touching a patient

Moment 2 - before clean/aseptic procedure

Moment 3 - after a procedure or body fluid exposure risk

Moment 4 - after touching a patient

Moment 5 - after touching a patient's surroundings

When thinking about the consequences for others if you became infected, which of the “5 moments of hand hygiene” do you intend to comply with most diligently during your work in the future when comparing them with each other?

Moment 1 - before touching a patient

Moment 2 - before clean/aseptic procedure

Moment 3 - after a procedure or body fluid exposure risk

Moment 4 - after touching a patient

Moment 5 - after touching a patient's surroundings

***Self-focus*** condition

When thinking about the consequences for yourself if you became infected, which of the “5 moments of hand hygiene” do you deem as most important when comparing them with each other?

Moment 1 - before touching a patient

Moment 2 - before clean/aseptic procedure

Moment 3 - after a procedure or body fluid exposure risk

Moment 4 - after touching a patient

Moment 5 - after touching a patient's surroundings

When thinking about the consequences for yourself if you became infected, which of the “5 moments of hand hygiene” do you intend to comply with most diligently during your work in the future when comparing them with each other?

Moment 1 - before touching a patient

Moment 2 - before clean/aseptic procedure

Moment 3 - after a procedure or body fluid exposure risk

Moment 4 - after touching a patient

Moment 5 - after touching a patient's surroundings

***Both*** conditions:

How comprehensible do you perceive the “5 moments of hand hygiene”?

1 2 3 4 5 6 7

Not at all comprehensible very comprehensible

How often do you comply with the recommend 30 seconds of disinfecting your hands?

Always mostly often times sometimes rarly never

How much would a reduction from 30 seconds to 15 seconds facilitate compliance with the 5 moments of hand hygiene?

How often would you comply with 15 seconds of disinfecting your hands?

Always mostly often times sometimes rarly never

Please describe your current working situation of the past 4 weeks using the following word pairs. Please choose the adjective that best describes it.

In general, my working situation in the past four weeks was

Pleasant unpleasant

full of conflict harmonious

easy difficult

frustrating inspriring

challenging threatening

We now present some general statements to you and ask you to indicate for each of the statements how good it describes you.

| does not describe me well  1 | 2 | 3 | 4 | 5 | 6 | describes me very well  7 |
| --- | --- | --- | --- | --- | --- | --- |
|  |  |  |  |  |  |  |

1. I often have tender, concerned feelings for people less fortunate than me.

2. Sometimes I don’t feel very sorry for other people when they are having problems.

3. When I see someone being taken advantage of, I feel kind of protective towards them.

4. Other people’s misfortunes do not usually disturb me a great deal.

5. When I see someone being treated unfairly, I sometimes don’t feel very much pity for them.

6. I am often quite touched by the things that I see happen.

7. I would describe myself as a pretty soft-hearted person.

In the last month, how often…

| never  1 | 2 | 3 | 4 | Very often  5 |
| --- | --- | --- | --- | --- |
|  |  |  |  |  |

1 …have you been upset because of something that happened unexpectedly?

2 …have you felt that you were unable to control the important things in your life?

3 …have you felt nervous and “stressed”?

4 …have you felt confident about your ability to handle your personal problems?

5 …have you felt that things were going your way?

6 …have you found that you could not cope with all the things that you had to do?

7 …have you been able to control irritations in your life?

8 …you felt that you were on top of things?

9 …you been angered because of things that were outside your control?

10 …have you felt difficulties were piling up so high that you could not overcome them?

We are now interested in some demographics.

Which occupational group do you belong to?

Nurses physician medical assistant other­­: ___________________

How long are you already working in your current job?

Less than 1 year

Between 1 and 5 years

Between 5 and 10 years

Between 10 and 20 years

Between 20 and 30 years

More than 30 years

How many days did you work during the past 4 weeks

On less than 10 days

Between 10 and 20 days

On more than 20 days

In which area do you work?

Internal medicine ward

Surgical medicine ward

Internal medicine ICU

Surgical medicine ICU

Other:______________________________

Are you actually taking care of COVID19 patients?

Yes No

Please indicate your gender

Male female diverse

You may leave a comment regarding this study here:

__________________________________________________________________________________________________________________________________________________________________________________________________________________________________________________________________________________________________________________________________________________________________________________________________________________________

This study is now finished. Thank you very much for your participation.

Again, If you have any questions please contact the principal investigator PD Dr. Claudia Sassenrath ([claudia.sassenrath@uni-ulm.de](mailto:claudia.sassenrath@uni-ulm.de)).

By clicking on the following link, you will be guided to a new Website where you can leave your email address there so that we can accord the voucher to you.
